# Perception of the non-dominant hand as larger after non-judgmental focus on its details

**DOI:** 10.1038/s41598-022-19919-6

**Published:** 2022-09-19

**Authors:** Ata Ghaderi, Elisabeth Welch

**Affiliations:** 1 Division of Psychology, Department of Clinical Neuroscience, Karolinska Institutet, 171 77 Stockholm, Sweden; 2 Stockholm Health Care Services, Region Stockholm, Stockholm Center for Eating Disorders, Wollmar Yxkullsgatan 27B, 118 50 Stockholm, Sweden; 3 Department of Clinical Neuroscience, Centre for Psychiatry Research, Karolinska Institutet, Norra Stationsgatan 69, 113 64 Stockholm, Sweden

**Keywords:** Psychology, Human behaviour

## Abstract

We investigated whether brief non-judgmental focus on the details of one’s non-dominant hand might lead to changes in perception of its size, and if such a change would be related to central coherence, body dissatisfaction, or how much participants liked their hand. After two pilot experiments (N = 28 and N = 30 respectively: Appendix 1), a within-subject experiment (N = 82) was conducted. Subjects were mainly university students. They were asked to rate the size of their non-dominant hand and how much they liked it, and the size of an external object (a X-box controller) on a visual-analog scale before and after focusing on their details for 5 min, as well as the size of another object (a calculator) before and after a 5 min long distraction task. After completing the tasks, they were asked to respond to a brief questionnaire on body dissatisfaction. A s significant interaction between time and factors (non-dominant hand, X-box controller and calculator) emerged (*F*(2, 78) = 6.41, *p* = .003). Participants rated their hand as larger after focusing on its details compared to baseline, and this change was significantly larger than those reported for the X-box controller. No significant change in how they liked their hand was observed, and contrary to the pilot experiments, the perceived change in size of the hand was not related to body dissatisfaction. The significant change in reporting of the size of the hand after focusing on its details seems to be an interesting finding, worth further replications.

## Introduction

Adequate perception of size is a necessary ability for successful interaction with our environment. The size of our body influences the perceived size of the world^1^, and it is an important determinant of what actions we perceive as possible, and how to execute them^2^. We often judge the size of different stimuli in various daily activities, without much conscious effort. As an example, lifting a mobile phone to answer a call includes a very fast judgment of the size of the phone to adjust the width of our grasp. Another example is how we adjust and shift the angle of our body when we pass through a narrow door opening, or through a crowd. We use different heuristics to make faster and more pragmatic decisions about metric such as size, weight, height and distance. As an example, our dominant hand seems to be a natural perceptual metric^3^, in the same way as the eye height is used to scale the height of objects on ground plane^4,5^.

Despite advances in our understanding of the processes that explain the relationship between our body representation and visual size perception e.g.,^1–3,6^, our understanding of basic processes involved in body size distortion and body image issues is limited. Problems related to perception of body, or body dissatisfaction have generally been investigated from a sociocultural, emotional, behavioral, or attitudinal perspective. An example of a behavioral perspective is body checking, which is a common behavior among people with body image concerns, body shape dissatisfaction, eating disorders, or body dysmorphic disorder^7–10^. Although these perspectives are important in understanding problems related to perception of body or body dissatisfaction, the role of some basic mechanisms involved (e.g., attention) are often ignored. It has been known since ancient times that extended viewing of a an object can bias one’s perception of other objects^11^, a phenomenon known as adaptation^12^. Recent studies have found that adaptation occurs for not only pictures of whole body but also isolated limbs, suggesting that such adaptation arises in high level mechanism that are specific for body parts, which is indicative of a presentation of bodies based on parts and their relationships^13^. Although many studies have investigated human perception and recently its role in body shape or body size misperception in light of visual adaptation (for a review see^12,14^), only a limited number of studies have investigated the role of attention in this context. Visual attention is a process involving the ability to orient to and sustain focus on a stimulus. There are three types of visual attention according to Carrasco^15^: The first one is spatial attention that can be overt or covert. The second form is featural attention, which is deployed covertly to various aspects of stimuli (e.g., motion direction or color), and the third is object-based attention which is related to the structure of the stimuli. Stephen and colleagues (2018) showed that visual attention mediated the relationship between body dissatisfaction and proneness to the body size adaptation effect^16^. However, simple featural attention to body fatness or sex typicality (rating fatness on a simple scale, or sex typicality in terms of femininity/masculinity) failed to show any influence of the magnitude of adaptation effect among subjects exposed to picture that were manipulated to look fatter or thinner than average^17^. In order to investigate the basic processes involved in the etiology and maintenance of mental disorders, mechanistic studies are required^18^. This is also applicable to predisposing conditions such as body dissatisfaction that increase the risk for later development of eating disorders. Socio-cognitive, emotional, attitudinal, and genetic risk factors of psychiatric disorders have received more attention in this endeavor than some other core processes such as attention. Although some exceptions exist with regard to visual attention^12–14,17,19^, the majority of studies on perception of body do not focus on the impact of prolonged mindful attention to details of the body. The role of attention in misperception of size or shape of specific body parts that might increase the risk for the emergence or maintenance of disorders that involve body dissatisfaction merits further research. It has been shown that patients with eating disorders devote more visual attention to body parts they find least attractive when viewing images of their own body, while they direct more attention to the most attractive parts of images of other women^20–22^. A better understanding of the role of attentional processes in perception of size might help us to devise efficient behavioral procedures for prevention and treatment of distorted body image which is both a risk factor and a maintaining mechanism in some psychiatric conditions such as eating disorders, and a source of distress and diminished quality of life.

At present, no studies have investigated the role of prolonged attention to details in the perception of size, irrespective of other cognitive and emotional variables. To gain a better understanding of the potential role of attention, we investigated whether our perception of size changes as a mere consequence of prolonged and detailed attention to details. Hence, through an experimental within-subject design, we asked study participants to report the size of a fairly neutral body part (their non-dominant hand) before and after 5 min of focus to its details.

**Figure 1:**
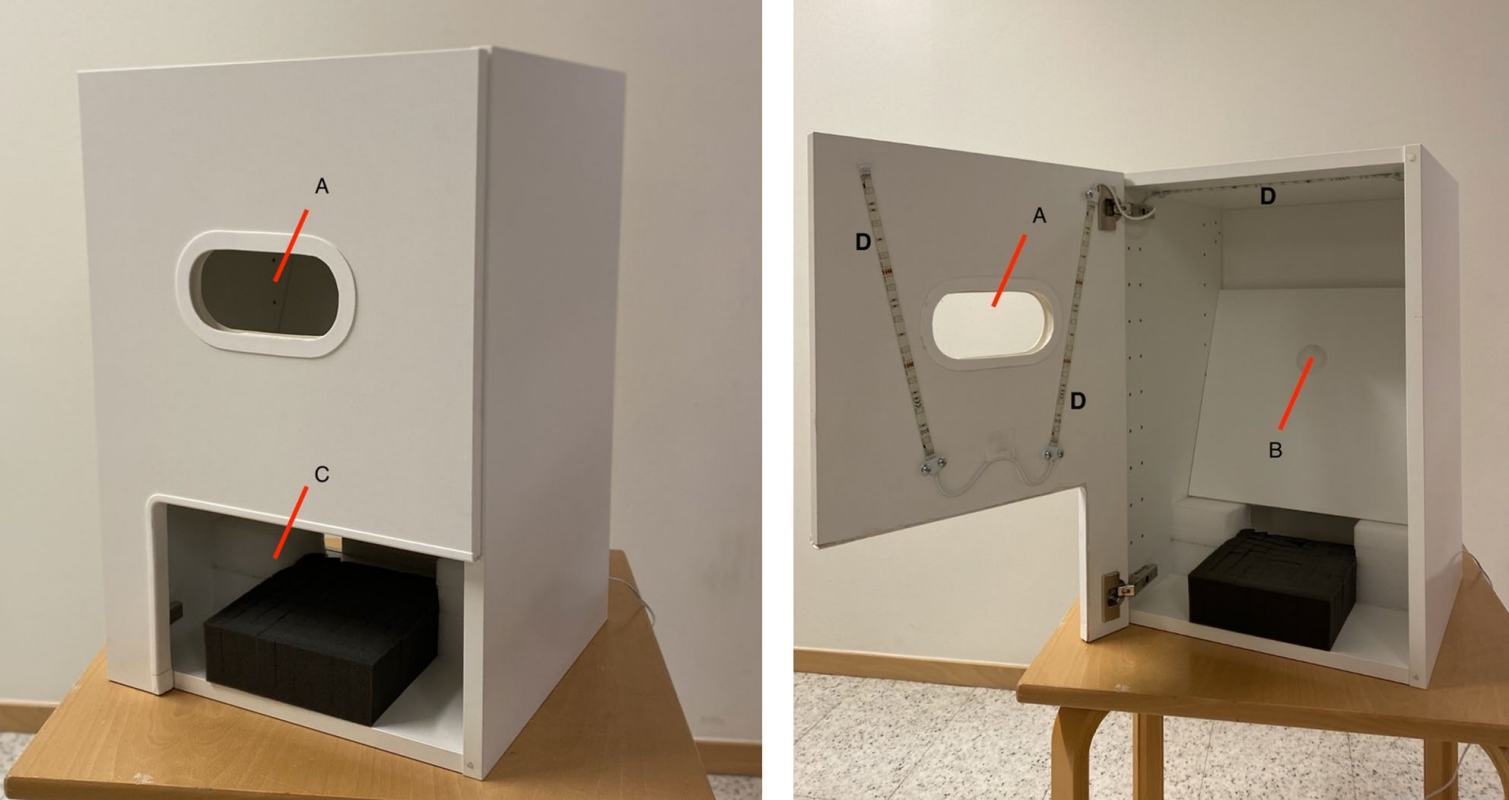
The box used for the experimental trial. Participants looked into the box to view their non-dominant hand, or other stimuli through (**A**), while their forehand touched the cabinet (left picture). LED strips inside the box (right picture) provide constant and uniform illumination. The non-dominant hand or objects were placed at a diagonal piece of wood (**B**). The gap at the lower front (**C**) was designed for inserting the non-dominant hand, and resting it on the (**B**).

The reason for choosing the non-dominant hand is that it can be easily isolated from other stimuli in the field of vision (e.g., by putting it into a specifically designed box: see Fig. 1), and the angle of viewing and the distance between eyes and hand can be easily controlled. In addition, most people do have a positive or neutral emotional stance toward their hands. Consequently, the non-dominant hand was considered an optimal body part for investigating the role of focus on details for perception of size.

Some researchers argue that our senses provide us with a rich experience of a detailed visual world^23,24^, and show that explicit reports of summary statistics underestimate the richness of the ensemble perception^24^. Focus on details might provide a wealth of explicit information and thus create new sets of contextual cues for altered judgment of the properties of stimuli including size. People seem to have a fairly constant view of the size of their body^3^ regardless of its precision (i.e., relation to actual size). However, based on clinical observations, we assume that when contextual cues for judgement of a specific body part changes toward more richness in information and details, its properties, including size might also be altered. We hypothesized that participants would experience their non-dominant hand as significantly larger after focused attention to its details compared to their initial estimation of its size. For experimental control, the participants rated the size of other physical objects before and after 5 min of focused attention to their details, or a few minutes of distraction. We hypothesized that the size of external objects would also be perceived as bigger as a consequent of focus on their details, while repeated ratings of objects without paying attention to their details would not be different from time to time. The perception of our body parts might also be affected by our emotional stance towards our body, and our inherent propensity to form meaningful and coherent wholes by integrating relevant pieces of information and drawing out meaning often at the expense of details (also called central coherence^25^). Therefore, we also asked the participant to rate how much they liked their non-dominant hand, and we investigated the relationship between central coherence and potential changes in the perceived size of the hand before and after focusing on its details. However, as no relationship with central coherence emerged in the pilot experiments, it was omitted. Finally, we investigated whether the potential changes in the perceived size of the hand were related to body dissatisfaction in general.

## Methods

This paper describes an experiment which was initially piloted and optimized in two pilot experiments. The study was approved by the Regional Ethics Board in Stockholm (Dnr. 2014/451-31/5). The methods, results and brief discussion of pilot experiment 1 and 2 are described in Appendix 1. Below, we present the methods of the main experiment, followed by results and discussion.

### Participants

A total of 82 participants (57.3% females) from three campus (Karolinska institutet, Stockholm University and Royal Institutet of Technology) with a mean age of 25.7 years (SD = 5.5) participated. They all had normal or corrected-to-normal vision. To investigate the test–retest reliability of the visual-analog ratings of the size of the hand, 24 additional participants (Mean age: 27.4 (SD = 6.7), age range 18–46 years old, and 50% females) were recruited and asked to rate the size of their non-dominant hand twice with 5 min in between the ratings.

### Procedure and instruments

Information about the study was announced on the campus using flyers on designated boards. As in the pilot experiments (Appendix 1), the study was announced as an investigation of how we perceive our body parts as a consequence of attention and various contextual effects (i.e., in vague terms) to minimize the risk of bias in how the participants might respond if they knew the specific study hypotheses. Those interested contacted the researchers who scheduled appropriate time for their participation. Upon arriving, they received information and instructions. All the instruction (verbal and in written form), with the exception of those related to greeting the participant, providing information about the study, and obtaining consent were pre-recorded on a computer using the software PsychoPy2 to standardize the procedures. All the ratings were done on the computer as well (A 13 inch MacBook Air, from 2016). Participants followed the verbal and written instructions provided on the computer, and completed the tasks successfully. The experiment was conducted in a designated test room at Karolinska Institutet. After providing consent, the participants were asked to remove rings, watches, etc., and to wash their hands, as in the pilot experiments. They were asked which one was their dominant hand, and the computer was then placed at comfortable reach of their dominant hand besides the cabinet (Fig. 1). The participants were instructed to find a convenient and relaxed position on a chair for looking into the opening of a cabinet (Fig. 1 depicting a standard white IKEA cabinet: depth = 38, breadth = 40, and height = 60 cm in size). The height of the table on which the cabinet was placed could also be adjusted to find a comfortable position for the participant. They were informed that different objects will be presented inside the cabinet twice, and they will be asked to provide ratings regarding their size, and that one of these objects will be their non-dominant hand. Participants were asked to use the opening of the cabinet (A in Fig. 1) to look at the objects, with their forehead touching the cabinet to ensure that the distance and lighting was consistent. The distance from the participants eyes to the hand or objects was 29 cm. The participants’ non-dominant hand could be inserted through the lower opening of the cabinet (C in Fig. 1), and placed on a diagonal board (B in Fig. 1) in the supinated position for viewing their palms and fingers. They were also instructed to keep their fingers fairly straight, but relaxed. Objects were also positioned on the same board using Velcro tape. Lighting was placed on the inside of the cabinet (D in Fig. 1) to provide constant and uniform illumination.

They were instructed then to start the experiment by clicking on any key on the computer. The experiment leader was in the room during the entire experiment to answer any question, replace the items inside the box, and help with potential technical problems, but was positioned outside of the participant’s field of vision to minimize distraction.

The external objects used in the pilot experiments were replaced by a X-box controller and a calculator, both of which are approximately of the same size as a hand (please see Appendix 2). They had varying shades across the surface and buttons, and details in different shapes and colors. The hand, the X-box controller, and the calculator were presented using a counterbalanced design to avoid order effects. After presenting each stimuli, the participants were asked to make a rating of its perceived size using a visual analog scale from Very small to Very large. The line on which the visual analog ratings were done was 10 cm long, providing ratings from zero (left size) to 100 (right side). Measures were programmed to be obtained to the nearest millimeter from the left endpoint (zero) of each line. In the hand condition, the participants were instructed to insert their hand into the cabinet, and make a rating of its size on the computer after 3 s of observing the hand. Then, they were asked to focus on their hand again following instructions to pay attention to different details of their hand in a factual, non-judgmental manner (texture, colors, blood veins, lines, ruggedness, etc.) for 5 min (M = 311 s including rating). Then they were asked to make another rating of the size of their hand. Participants were also asked to provide a rating of how much they liked their hands on a visual analog scale from “Do not like it all” to “Like it very much” after each rating of the size of their non-dominant hand. The X-box controller condition was similar to the hand condition. They provided ratings of its size before and after focusing on its details. The calculator was the second control object. After rating its size, participants listened to a text on Writing systems and its development (“https://en.wikipedia.org/wiki/Scripting_language”) for 5 min. Any references to numbers, distances, mass, size, etc. was removed from the text. No ratings of Liking were collected for external objects.

After completing the experimental tasks, participants completed the questionnaire on demographics, and the Body Shape questionnaire (BSQ)^26^. The BSQ provides a measure of body dissatisfaction and possesses good psychometric properties. The internal consistency of the BSQ in this experiment was 0.88. The participants were then debriefed, and received two cinema tickets for their participation.

### Statistical analysis

To investigate whether the changes within each condition across assessment points were significantly different from each other, the interaction between time and conditions were investigated using analysis of variance (ANOVA), in addition to the time and condition effect. Means, standard deviations and magnitude of change within each condition are presented along with the effect size of the change using Cohen’s *d*. The magnitude of change in rating of the hand before and after focus on its details was compared to the change in ratings of the X-box controller before and after focus on its details by means of *t*-test. The relationship between change in the size of the hand, and body dissatisfaction was investigated using Pearson correlation coefficient. All the statistical tests are two-tailed unless otherwise specified. All the analyses were performed in SPSS.

The within group effect sizes for change in size of the hand in pilot experiment 1 and 2 were 0.70 and 0.48. Accordingly, for a power of 0.99, with p < 0.05, we would need at least 29 to 59 participants in the experiment. To guard against potential attrition or incomplete data, as well as sufficient power for interaction between time and conditions, at least 80 participants were planned to be recruited.

### Ethics approval and consent to participate

The studies were approved by the Regional Ethics Board in Stockholm (Dnr. 2014/451-31/5). Informed written consent was obtained from all the participants, and the study was conducted in accordance with relevant guidelines and regulations.

## Results

A repeated measures ANOVA exploring the effect of viewing time (baseline vs after focused attention or distraction), condition (non-dominant hand, X-box controller and calculator), and their interaction (Time X Condition) on ratings of size was conducted. ANOVA showed a significant time effect (*F*(1, 79) = 4.90, *p* = 0.03, *η*_*p*_^2^ = 0.06), a non-significant effect of condition ((*F*(2, 158) = 1.19, *p* = 0.31, *η*_*p*_^2^ = 0.03), and a significant interaction between time and conditions (non-dominant hand, X-box controller and calculator) (*F*(2, 158) = 6.45, *p* < 0.001, *η*_*p*_^2^ = 0.14). The mean change in the size of the hand was significantly larger than the mean change in the size of the X-box controller (*t*(79) = −2.21, *p* = 0.030, Cohen’s *d* = 0.25)*.* Participants rated their hand as bigger after focusing on its details compared to baseline (Table 1).

**Table 1:** The mean and standard deviation of ratings of the non-dominant hand and external objects, and the effect size (Cohen’s d) of the change between baseline rating and the rating after focusing on details of the hand or the X-box controller, or listening to the distraction task (calculator condition).

	Baseline	After focusing	t-test	Cohen’s d
The non-dominant hand	52.5 (16.1)	59.7 (16.8)	*t(79)* = −3.52, *p* < 0.001	0.39
X-box controller	52.6 (15.2)	55.3 (16.2)	*t(79)* = −1.57, *p* = 0.121	0.18
Calculator	56.5 (16.4)	54.8 (16.3)	*t(81)* = 1.28, *p* = 0.205	0.14

**Figure 2:**
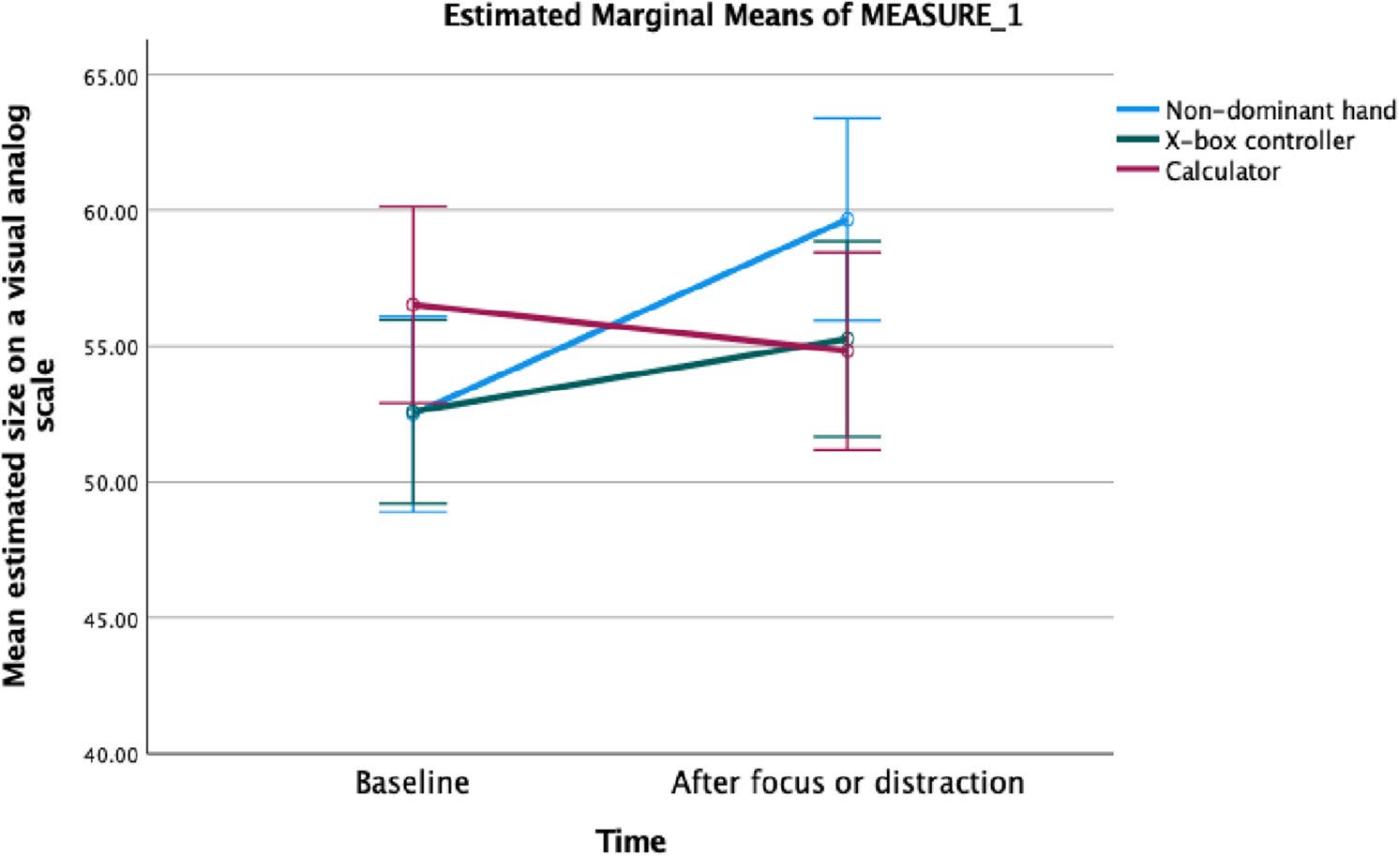
Graphic illustration of the mean estimated size of the non-dominant hand, the X-box controller and the calculator at baseline and after focus on the details or a distraction task. The error bars represent 95% confidence interval around the means.

The mean ratings and 95% confidence interval around the means are also presented in Fig. 2.

Baseline scores of how much they liked their hand (*M* = 74.3, *SD* = 17.8) was not significantly different (*t*(79) = 0.59, *p* = 0.557, Cohen’s *d* = 0.07) from rating after focusing on its details (*M* = 72.9, *SD* = 19.8).

None of the correlations between the change in the rating of the hand size and likability (*r* = −0.17, *p* = 0.14) or body dissatisfaction (*r* = −0.004, *p* = 0.97) were statistically significant.

**Figure 3:**
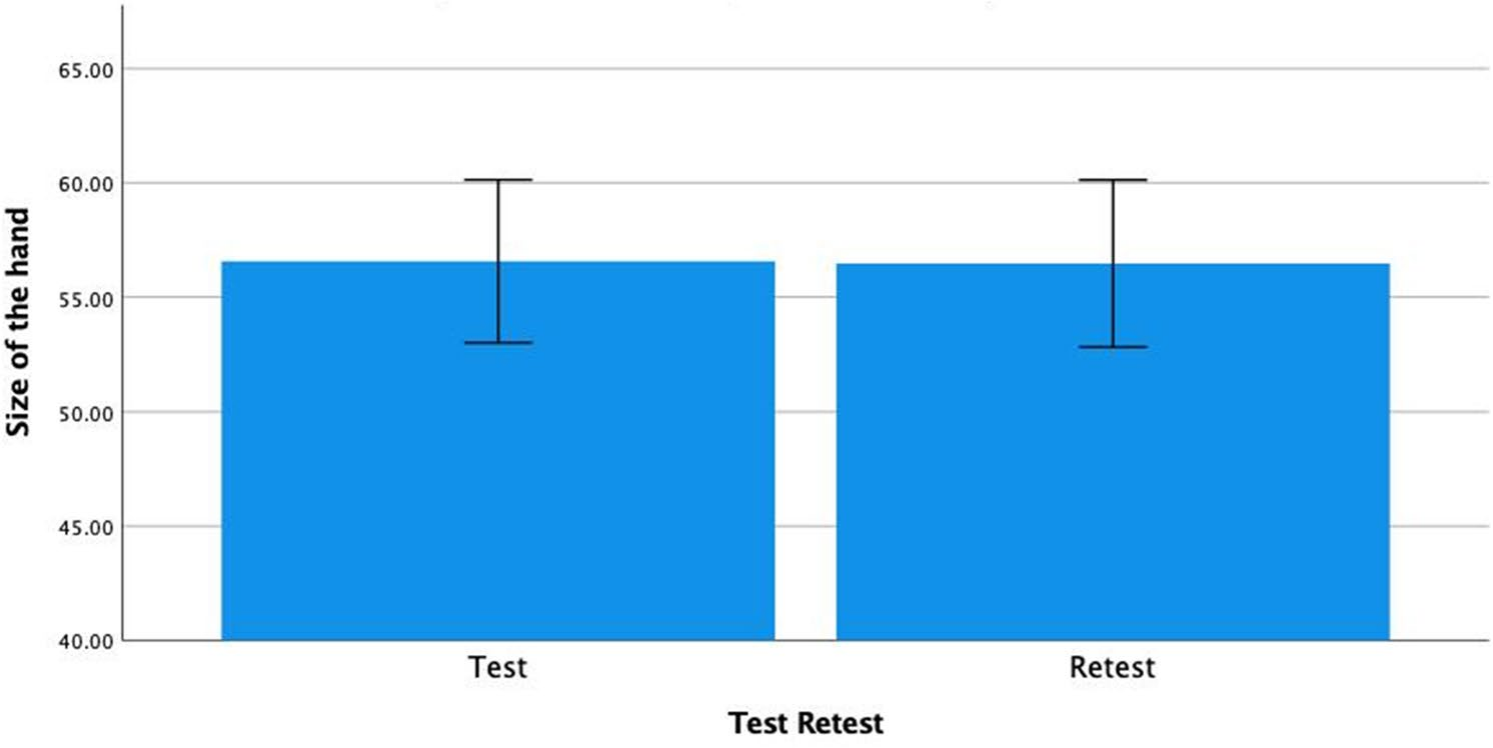
Bar graph of the first and second rating of the size of the hand on a visual analog scale (means and 95% CI of the means).

To establish the test–retest reliability of the visual-analog ratings of the non-dominant hand, the same distraction task as in the experiment was used with a convenience sample of university students (N = 24, age: 27.4 (SD = 6.7), age range 18–46 years old, and 50% females). The first rating (M = 56.6, SD = 8.4) was not significantly different (F(1, 23) = 0.08, p = 0.78) from the second rating (M = 56.5, SD = 8.7). The mean and the 95% CI around the mean is presented in Fig. 3. The test–retest reliability was high (r = 0.98, p < 0.001).

## Discussion

The experience gained in pilot experiments (Appendix 1) helped to optimize the current experiment. The main outcome (the change in the perceived size of the hand before and after focusing on its details) was statistically larger than the changes in control conditions similar to pilot experiments. The ratings of how much the participants “liked their hand”, were not significantly different before and after focusing on its details. In addition, the reported change in the size of the hand was not significantly related to body dissatisfaction, in contrast to the first pilot experiment. The magnitude of effect for change in the size of the non-dominant hand was close to the one observed in pilot experiments, indicating a potentially robust effect.

To summarize, the rating of size of the non-dominant hand seems to change after focusing on its details. Longo and Haggard (2010) suggested that a combination of a stored implicit body model and immediate proprioceptive afferent signals are necessary for perceiving the external spatial location of the hand through position sense. They showed that the body model of hand is distorted in a stereotyped way across individuals^27^. Further work using their paradigm showed that magnitude of this distortion is significantly different depending on whether the subjects are blindfolded or were able to see where they were pointing^28^. They concluded that vision may modulate the representations of body size and shape, even when it is not informative^28^. In other words, they suggest profound interactions between vision and somatosensation in general and proprioception in particular. It remains to be investigated in what ways prolonged attention to details affects the perception of size. Would prolonged non-judgmental focus to details affect the implicit body model of hand through down-, or upregulation of the already known distortions of the implicit hand map? Furthermore, the role of rapid shifts of involuntary attention due to peripheral pre-cues, leading to a phenomenon called attentional repulsion effect might also offer some insights into the mechanisms involved in perception of size. In a recent study^29^, attentional repulsion effect was suggested to reflect a localized, symmetrical warping of visual space that impacts both shape and location of target stimuli. Sustained attention may alter the receptive field of visual cells, and thereby change the perception of size and location^30,31^.

Although refraining from detailed scrutiny of the body and body parts has been implicated in some clinical approaches such as those involved in addressing body checking in transdiagnostic cognitive behavior therapy for eating disorders^32^, the findings in the current study are to our knowledge the first experimental evidence of potential effect of prolonged attention to details and change in the perception of size of a body part. Although the current experiment is not a case of visual adaptation for understanding body size misperception^12,14^, the insights from studies on visual adaptation or cross adaptation might be useful in future studies of the role of attention on perception of size. Prolonged exposure to stimuli with certain direction, orientation or motion leads to biased perception of subsequent stimuli. For example, watching a series of pictures of thin bodies makes subjects judge subsequent normal bodies as somewhat larger, while exposure to picture of obese people biases the perception of subsequent normal size bodies toward thinner. Attention and details of the stimulus properties complicate the picture, but it might provide some clues to the underpinning of the observed biases. Pestilli and colleagues (2007) suggested that attention optimizes performance by increasing contrast sensitivity for attended stimuli and neural responses to them^33^. In an experimental study of the role of attention and adaptation for contrast sensitivity, they found that attention increases stimulus salience, and it can overcome adaptation to restore contrast sensitivity^29^.

Future studies might benefit from investigating contrast sensitivity, attention and potential role of adaptation in understanding the processes that contributed to our findings. In a recent summary of findings on visual adaptations as a mechanism for body-size and body-shape misperception, Brooks and colleagues have provided several viable options for pursuing the potential of this mechanism to prevent and treat misperception of body size or shape^14^. It has been suggested that behavioral interventions may benefit from using principles from visual adaptations by helping patients with for example anorexia nervosa spend time viewing ideal bodies versus normal bodies and to investigate how it affect their own body image^12^, as such assignments might help patients reduce their exposure to ideal bodies and be more accepting of weight gain. However, Mohr and colleagues showed that adaptation to thin images was therapeutic for healthy subjects but not for those with anorexia or bulimia nervosa^34^. We need a better understanding of the determinants and processes involved in body dissatisfaction and body size or shape misperception before we can devise effective interventions.

Body dissatisfaction may be initiated and maintained through a vicious cycle. This cycle may emerge as a consequence of several processes. Examples of such processes are: a tendency to devote more visual attention to body parts that are perceived as least attractive among those with body dissatisfaction or eating disorders^20–22^, in combination with prolonged attention to details that might change the perception of size as shown in the current study, and a negative emotional stance toward these body parts and related cognitions.

In terms of a theoretical model, it is plausible to assume that mere attention to body parts in a focused way might alter the perception of the size of the body in a way that evokes dissatisfaction, anxiety and other negative feelings. Such negative cognitions and emotions might contribute to increased scrutiny of those specific body parts in a vicious cycle. Body ideals and emotional, as well as cognitive stance of the individual would most probably play an important role in such a model, which needs further investigation in studies where all of these variables and their interactions are studied.

Although some studies have shown that contextual cues such as exposure to pictures of overweight or thin individual^19^ impact the participants’ perception of “normal” body size, such an exposure does not affect body dissatisfaction. While the second pilot showed a relationship between the change in the perception of size of the non-dominant hand and how much the participants “liked their hands” before and after exposure to its details, such a relation did not emerge in the main experiment. Similarly, no significant change in how much they liked their hand before and after focusing on its details emerged, unlike what we found in the second pilot experiment. It remains an empirical question whether the sensitivity of the ratings for the visual analog scales was lower in the experiment compared to the pilot experiment due to use of a shorter VAS line ^10cm^ for making the ratings compared to the pilot experiments 1 and 2 (14.3 and 13 cm). However, lower ratings of likability after focusing on details are somewhat unexpected as our hands often are one of the most appreciated or at least neutral parts of our body^35^, probably due to their importance for everyday functioning. Future independent replications are crucial to determine whether a true change in likability occurs as a result of non-judgmental focus on details.

A significant limitation of the study is the comparison of an animate stimulus (i.e., subject’s own hand) with inanimate stimuli (X-box controller and calculator). The found differences in this study might be a consequence of comparing an animate versus an inanimate stimulus. Thus, future replications should include an animate control stimulus to investigate this possible alternative hypothesis. Use of a visual-analog scale is another significant limitation of the study. Future studies may also add more advanced ratings such as computerized pictures of the stimuli in the experiment that can be scaled up and down to make ratings after each exposure, to provide a basis for comparison to actual size. However, depictive versus metric methods might reflect different aspects of our perception^27^. Although visual analog scales have some inherent limitations, they showed good stability in repeated ratings of stimuli that were not manipulated (e.g., Tape dispenser in pilot experiment 1, and calculator in the main experiment), and very high test–retest reliability. Nevertheless, additional systems for rating of the size should be developed, to increase the reliability and sensitivity of the ratings. In addition, refining the rating system increases not only the reliability, but also the validity of future studies. In future replications, more complex control stimuli than those used in the current study should be used. Our hand palms and fingers are tremendously complex in terms of details (structure, color, shape, etc.). Control objects should also possess such complexity to make a fair comparison. In addition, a recent study found that implicit perceived width of the hand was dependent on the orientation of the hand (upright versus right orientation), but overestimation in perception of length of the hand only occurred for dorsum and not the palm^36^. The orientation of the hand as well as whether the potential change in perception of size is isotropic or not should be considered in future replications.

Furthermore, it would be important to investigate whether attention to details might also affect the perception of the size of other body parts that the participants perceive as least and most attractive. The participants’ attitudes regarding common fashion and thinness ideal, as well as attitudinal and behavioral striving to approach or to distance themselves from such ideals should also be assessed to put the findings into context. Another important step in future replications, is to include several indices of central coherence, in contrast to a simple measure, as in the current study. Likewise, although the BSQ is an established measure of body dissatisfaction, most of the research on its reliability and validity has been done on females. The BSQ does not capture some aspects of body dissatisfaction such as muscle dysphoria that are more prevalent among males than females, and thus might constitute a source of bias in the study. Finally, response bias in perceptual learning is a known phenomenon^37^. The design of our experiment cannot completely rule out the presence of any response bias. However, given the low number of exposures to each stimulus there should be very small room for increased precision in sensory judgement as a source of bias, and the direction of change across the stimuli and measurements is not suggestive of response bias either.

## Conclusions

Despite the limitations, the experimental within-subject design of the study, and the consistency of the main finding across the pilots and the main experiment suggest an effect (perceiving the non-dominant hand as bigger after focusing on its details) that justifies further investigations to understand the mechanisms that account for the effect, and its implications.

## Supplementary Information

Supplementary Information 1.

Supplementary Information 2.

## Data Availability

Data will be made available upon request. Please contact the corresponding author.
