# Supplementary material for: Perception of the non-dominant hand as larger after non-judgmental focus on its details
Source: Sci Rep. 2022 Sep 19;12:15670. doi: 10.1038/s41598-022-19919-6 (PMC9485221; doi:10.1038/s41598-022-19919-6)
Supplement: Supplementary file 1 — Supplementary Information 1. [file 41598_2022_19919_MOESM1_ESM.docx]

# Methods

**Pilot experiment 1**

**Participants.**

A convenient sample of university students (n=28, 67.9% females) with a mean age of 26.0 years (SD=5.8) participated. They all had normal or corrected-to-normal vision.

**Procedure.**

Information about the study was announced on the campus using flyers on designated boards. The study was announced as an investigation of how we perceive our body parts as a consequence of attention and various contextual effects (i.e., in vague terms) to minimize the risk of bias in how the participants might respond if they knew the study hypotheses. Those interested contacted the second author and made an appointment to participate. They received information about the study and provided written consent, upon which they were instructed to wash their hands, remove rings or other ornaments from their non-dominant hand, to find a convenient and relaxed position on a chair for looking into the opening of a cabinet (Figure 1). The height of the table on which the cabinet was placed could also be adjusted to find a comfortable position for the participant. They were informed that different objects will be presented inside the cabinet twice, and they will be asked to provide ratings regarding their size, and that one of these objects will be their non-dominant hand. Participants were asked to use the opening of the cabinet (A in Figure 1) to look at the objects, with their forehead touching the cabinet to ensure that the distance and lighting was consistent. The participants’ non-dominant hand could be inserted through the lower opening of the cabinet (C in Figure 1), and placed on a diagonal board (B in Figure 1) in the supinated position for viewing their palms and fingers. Objects were also positioned on the same board using Velcro tape. Lighting was placed on the inside of the cabinet (D in Figure 1) to provide constant and uniform illumination.

The order in which the objects (the non-dominant hand, and a tape dispenser) were presented was randomized. Participants were also asked to provide a rating of how much they liked their hands on a visual analog scale from “Do not like it all” to “Like it very much” after each rating of the size of their non-dominant hand. Finally, they completed two brief questionnaires and a test measuring central coherence. The experiment leader followed a study protocol to standardize the procedures during the experiment.

**Instruments.** To standardize the procedure and control for egocentric distance and location, a standard white IKEA cabinet (depth=38, breadth=40, and height=60 cm in size) was constructed for the purpose of the study (Figure 1). Participants responded to a few of background questions (age, gender, the last time a vision check was done, use of any vision correction aids such as lenses or contacts), as well as two questions on low mood, and on concentration on the task on a visual analog scale after completing the experimental task. They also completed the 8-item version of the Body Shape Questionnaire (BSQ) that possesses good psychometric qualities (1) to assess their body dissatisfaction, and the Group Embedded Figures Test (GEFT) (2, 3) to assess their central coherence. The size of the hand and objects were rated on a 14.2 cm visual analog scale from very small to very large on a separate sheet of paper for each rating in the pilot trial. The ratings were measured to the nearest millimeter from the left end of the line. There is a large body of research on depictive versus metric methods, and their relation to implicit versus conscious body image (4, 5). Given the relationship of metric methods to somatosensory body representations (5), a visual analog scale was considered a simple and good enough choice as the aim was to obtain a metric measure of perceived size which is inherently related to somatosensory body representation of each person, and not its accuracy in relation to the objective size of the hand. The objective size of the hand was not measured.

The instructions for focusing on the details of the hand were provided in a standardized text that was read by the experiment leader to help the participants focus on details of their hand in a factual, non-judgmental manner (texture, colors, blood veins, lines, ruggedness, etc.). Likewise, the instructions for focusing on the control object (a tape dispenser) were standardized to match the length of the instructions for focusing on the hand, with appropriate references to its details.

**Statistical analysis.** To investigate whether the changes within each factor across assessment points were significantly different from each other, the interaction between time and factors were investigated using analysis of variance (ANOVA), in addition to the time and condition effect. Descriptive statistics and magnitude of change within each condition are presented along with the effect size of the change using Cohen’s *d*. The magnitude of change in rating of the hand before and after focus on its details was compared to the change in ratings of a Tape dispenser before and after focus on its details by means of a planned *t*-test. The relationship between change in the size of the hand, and body dissatisfaction was investigated using Pearson correlation coefficient. All the analyses were performed in SPSS.

The relationships between the change in size ratings and central coherence, or body dissatisfaction were investigated using Pearson’s correlation coefficient. *All the statistical tests are two-tailed unless otherwise specified.*

**Results**

In the ANOVA, a significant interaction between the time and conditions (non-dominant hand versus tape dispenser) emerged (*F*(1, 27)= 5.18, *p*=.031). The mean change in the size of the hand was significantly larger than the mean change in the size of the tape dispenser (*t*(27)= -2.27, *p*=.031, Cohen’s *d*=0.43). The time effect in ANOVA was non-significant (*F*(1, 27)= 0.61, *p*=.44), but the condition effect was significant (*F*(1, 27)= 6.02, *p*=.02).

The magnitude of the effect of change in ratings of hand before and after focus on its details was virtually large, while the change in the ratings of the tape dispenser was small (Table A1). Participants were also asked to rate how much they “liked their hand” on each occasion. Although they reported a slightly lower rating of how much they liked their hand after focusing on its details (*M*=75.7, *SD*=39.4) compared to baseline (*M*=83.1, *SD*=30.0), the difference was not significant (*t*(27)=1.87, *p*=.072, Cohen’s *d*=0.35).

Body dissatisfaction measured by the BSQ was significantly related to change in the ratings of the hand size (*r*=.39, *p*=.04). More pronounced body dissatisfaction was related to a larger discrepancy in the repeated ratings of the non-dominant hand. The total score of the Group Embedded Figures Test was not significantly related to the discrepancy in the ratings of the size of the hand (*r*=.11, *p*=.60). As central coherence and body dissatisfaction might also interact to influence size inflation vulnerability, the association between the interaction term of central coherence and body dissatisfaction to change in perception of size of the hand was investigated. The correlation was negligible and non-significant (*r*=.014, *p*=.47).

We found no significant correlations between how much they liked their hands and ratings of its size at each occasion, or change in ratings of its size.

Table A1

The mean and standard deviation of rating of the non-dominant hand and tape dispenser, at baseline and after focusing on details, along with the effect size of the change (Cohen’s *d*) in Pilot experiment 1.

|  | Baseline | After focusing | Cohen’s d |
| --- | --- | --- | --- |
| The non-dominant hand | 67.4 (19.7) | 83.3 (21.4) | *0.70* |
| Tape dispenser | 70.9 (16.7) | 73.6 (26.5) | *0.10* |

**Discussion**

As hypothesized, the non-dominant hand was perceived as larger after a few minutes of non-judgmental focus on its details. This is an interesting finding in need of replication. Such an effect was not present for the tape dispenser. A potential reason for lack of effects for the tape dispenser might be its simple structure and fewer details compare to the palm and fingers of a hand with all the shifts in color, texture, lines, muscles, etc. The precision in rating the size of the same object across time on the visual analog scale in the study was not known. For refining the experiment and to put the results into context, we also realized the importance of having an additional object to rate twice: once at baseline, and then after a few minutes of distraction. All the outcomes should be interpreted with caution given the low sample size and insufficient power in the analyses.

**Pilot Experiment 2**

The second pilot was a replication of the first pilot, with improved methodology in terms of increased standardization of the procedure.

**Participants.**

A total of 30 university students (66.7% females) with a mean age of 24.6 years (SD=6.5) participated. They all had normal or corrected-to-normal vision, and received a cinema ticket for their participation in the study.

**Procedure and instruments.**

Participants were recruited in the same way as in the pilot trial, through flyers on designated boards at the campus. Interested participants contacted a research assistant who provided information about the study, and obtained written consent. The same procedure as in the pilot trial was used. However, the participants completed the demographic questionnaire before working through the following three conditions: 1) Ratings of the size of their non-dominant hand, and how much they like their hand before and after four minutes of guided focus on its details on separate pieces of paper using a 13 cm long visual analog scale (VAS rating). The change in the length of the line compared to the pilot trial (14.2 cm) was initially unknown and an unexpected consequence of saving Microsoft Word documents as pdf-files before printing. The first rating of the size of the hand was done after inserting their hand into the cabinet, putting it on the board (B in Figure 1 in the main article) and looking at it for three seconds when their forehead was in contact with the cabinet (A in Figure 1). The participants were instructed to sit in a comfortable position by adjusting the height of the table, and the chair to be able to put their hand on the predetermined spot (B in Figure 1) and rest their forehead on the cabinet in a relaxed manner to face the palm of their hand and their fingers without moving their hands during the experiment. They were also instructed to keep their fingers fairly straight, but relaxed. Verbal instructions on what to focus on the palm and fingers were pre-recorded to standardize the procedure to a higher extent compared to the first pilot. 2) Ratings of the size of a large pencil sharpener before and after four minutes of guided focus on its details. Similarly, the instructions were pre-recorded for this condition. Both the instructions (for focusing on hand, and the pencil sharpener) were each 4 minutes and 2 seconds long. 3) Ratings of the size of a tape dispenser before and after four minutes of reading a text about the sun and planets. The text was taken from <http://sv.wikipedia.org/wiki/Solen> (in English: https://en.wikipedia.org/wiki/Sun). It was slightly simplified to increase its readability.

Separate sheets of paper to make the VAS ratings were presented one by one on the side of the cabinet on the table to make sure they could use their dominant hand. The experiment was completed in a sound-proof room. As soon as a rating was done, the paper was removed by the experiment leader to avoid interference with the next rating.

The order of conditions was randomized to prevent potential order effects. After running these three conditions, the participants completed the GEFT (3), and the BSQ (1). Participants were debriefed about the study and were asked to not discuss it with others until the project was completed.

The same instruments as in the pilot trial were used.

**Statistical analysis.** The same analyses as in the first pilot experiment (i.e., ANOVA, t-test, Pearson’s r, and Cohen’s d) were used.

**Results**

In ANOVA, a significant interaction between time and factors emerged (*F*(2 , 54)=6.72, *p*=.002). The time effect was not significant (*F*(1 , 27)=0.001, *p*=.98), while the overall difference between the conditions were significant (*F*(1 , 54)=6.72, *p*=.002). The mean change in the size of the hand was significantly larger than the mean change in the size of the tape dispenser (*t*(27)= -2.27, *p*=.031, Cohen’s *d*=0.43).

The magnitude of the within condition change and descriptive data are presented in Table A2. Notably, after a few minutes of distraction and listening to a text about stars and planets, they rated the tape dispenser as significantly smaller compared to baseline (Table A2).

Unlike the first pilot trial, the change in the rating of the size of the hand was neither significantly correlated with body dissatisfaction (*r*=-.09, *p*=.63), nor the measure of central coherence (GEFT: *r*=.005, *p*=.98).

The results of the first pilot experiment were also replicated in terms of no significant correlations between how much they liked their hand and ratings of its size at each occasion (*r*=.27, *p*=.16, and *r*= -.13, *p*=.52, respectively). Finally, we found no significant relationship between change in ratings of size and change in how much they liked their hand (r=.20, p=.30).

Table A2

The mean and standard deviation of rating of the non-dominant hand and external objects, and the effect size of the change in rating from baseline to after focusing on details or distraction task in Pilot experiment 2.

|  | Baseline | After focusing | Cohen’s d |
| --- | --- | --- | --- |
| The non-dominant hand | 72.7 (23.0) | 83.8 (24.9) | *0.48* |
| Tape dispenser | 66.8 (25.5) | 56.6 (22.6) | *0.45* |
| Pencil sharpener | 70.8 (20.8) | 74.4 (31.5) | *0.16* |

**Discussion**

Most of the findings from the first pilot experiment were replicated, with the most crucial ones being the increase in ratings of the hand size and reduction in how much they liked it after focusing on its details. Interestingly, the tape dispenser was perceived as smaller after a few minutes of listening to a distracting text about the sun and stars. In the debriefing of the participants after the experiment, quite a few participants mentioned that they felt overwhelmed by the notion of gigantic sizes, mass, time and distances in the text (e.g., “Its diameter is about 1.39 million kilometers …” or “It formed 4.6 billion years ago…”). Although the text was intended as a distracting task, it probably contributed to a sense of perceiving everything else as smaller than it is in the context of incredibly large numbers mentioned in the text. This is known as “awe” in the literature, defined as an emotional response to perceptually vast stimuli that is beyond one’s usual frame of reference (6, 7). It turned out that the distraction strategy failed, and a new replication with a more neutral and distracting task was required. As neither in this experiment, nor in the first pilot we found a relationship between the measure of central coherence and changes in the ratings of the hand size, it might be plausible to conclude that such a relationship might not exist, or at least not be a strong one. Thus, data did not justify further investigation of this relationship with the specific measures used in this study. The findings were inconsistent in relation to body dissatisfaction. While a correlation was found in the first pilot experiment, it was basically non-existent in the second pilot experiment. This inconsistency called for further investigations. It remained unclear why attention to detail seem to influence ratings of the hand, but not an external object (the pencil sharpener in this study). It was concluded that a more complex object should be used in the next experiment to ascertain whether the level of complexity might be an important dimension. Furthermore, a more adequate distraction task should be used, and the entire procedure (i.e., the administration of the experiment and ratings) should be standardized by pre-recording the instructions and enabling ratings on a computer screen. As noted in the first pilot, all the outcomes should be interpreted with caution given the low sample size and insufficient power in the analyses. The next experiment need to be based on adequate power analysis and thus sufficient sample size.

**References**

1. Welch E, Lagerstrom M, Ghaderi A. Body shape questionnaire: psychometric properties of the short version (BSQ-8C) and norms from the general Swedish population. Body Image. 2012;9(4):547-50.

2. Witkin HA, Oltman PK, Raskin E, Karp SA. A manual for the embedded figures tests. Palo Alto, CA: Consulting Psychologists Press; 1971.

3. Carter H, Loo R. Group Embedded-Figures Test: psychometric data. Percept Mot Skills. 1980;50(1):32-4.

4. Longo MR, Haggard P. An implicit body representation underlying human position sense. Proc Natl Acad Sci U S A. 2010;107(26):11727-32.

5. Longo MR, Haggard P. Implicit body representations and the conscious body image. Acta Psychol (Amst). 2012;141(2):164-8.

6. Keltner D, Haidt J. Approaching awe, a moral, spiritual, and aesthetic emotion. Cogn Emot. 2003;17(2):297-314.

7. Piff PK, Dietze P, Feinberg M, Stancato DM, Keltner D. Awe, the small self, and prosocial behavior. J Pers Soc Psychol. 2015;108(6):883-99.
