# Supplementary figures and images for: Perception of the non-dominant hand as larger after non-judgmental focus on its details
Source: Sci Rep. 2022 Sep 19;12:15670. doi: 10.1038/s41598-022-19919-6 (PMC9485221; doi:10.1038/s41598-022-19919-6)

Appendix 2

The X-box controller and the calculator used in the study.


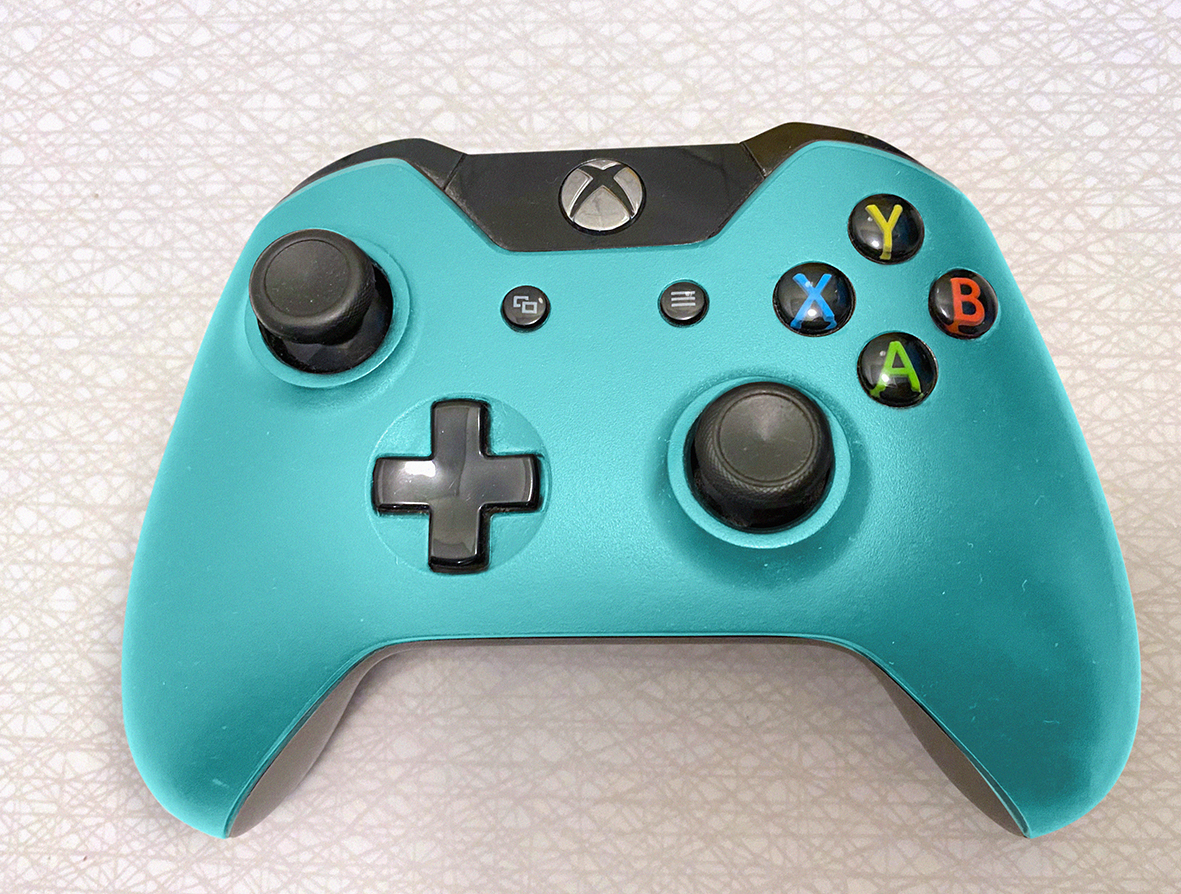

Supplement: Supplementary file 2 — Supplementary Information 2. [file 41598_2022_19919_MOESM2_ESM.docx]
